# Exploring the Role of Azurin from the Endophytic Bacterium *Pseudomonas* sp. OHS18 Through the Phenotypic Characterization of a Δ*azu* Mutant

**DOI:** 10.3390/microorganisms14071499

**Published:** 2026-07-09

**Authors:** Giulia Semenzato, Veronica Ghini, Valentina Pecchioli, Marta Iozzo, Giorgia Molesini, Francesco Imperi, Alberto Bernacchi, Giovanni Emiliani, Giovanni Stefano, Luciana Renna, Sonia Coves Mora, Elisa Masi, Renato Fani

**Affiliations:** 1 Department of Biology, University of Florence, 50019 Sesto Fiorentino, Italy; giuliasemenzato@cnr.it (G.S.); alberto.bernacchi2@unibo.it (A.B.); giovanni.stefano@unifi.it (G.S.); 2 Institute for Sustainable Plant Protection, Italian National Research Council, 50019 Sesto Fiorentino, Italy; giovanni.emiliani@cnr.it; 3 Department of Chemistry, University of Florence, 50019 Sesto Fiorentino, Italy; veronica.ghini@unifi.it (V.G.); valentina.pecchioli@unifi.it (V.P.); 4 Department of Experimental and Clinical Biomedical Sciences, University of Florence, 50134 Florence, Italy; marta.iozzo@unifi.it; 5 Department of Science, University of Roma Tre, 00146 Rome, Italy; giorgiamolesini@gmail.com (G.M.); francesco.imperi@uniroma3.it (F.I.); 6 National Biodiversity Future Center (NBFC), 90133 Palermo, Italy; 7 IRCCS Fondazione Santa Lucia, 00179 Rome, Italy; 8 Department of Biological, Geological and Environmental Sciences (BiGeA), University of Bologna, 40126 Bologna, Italy; 9 Department of Bioscience, Biotechnology and Environment, University of Bari Aldo Moro, 70121 Bari, Italy; luciana.renna@uniba.it; 10 Department of Agricultural, Food, Environmental and Forestry Sciences and Technologies (DAGRI), University of Florence, 50019 Sesto Fiorentino, Italy; scoves@mbg.csic.com (S.C.M.); elisa.masi@unifi.it (E.M.); 11 Biological Mission of Galicia, Spanish National Research Council (CSIC), 36143 Pontevedra, Spain

**Keywords:** blue proteins, redox homeostasis, phenotype microarray, NMR, plant–bacteria interaction

## Abstract

Azurin is a promising antitumor agent that selectively enters cancer cells and inhibits tumor progression. It is also known to participate in cellular processes involving single-electron transfer, including protection against oxidative stress, anaerobic respiration, and denitrification. However, the physiological role of azurin remains poorly understood. In this work, a multifaceted phenotypic characterization of an azurin-deficient mutant (Δ*azu*) of the plant endophytic bacterium *Pseudomonas* sp. OHS18 was performed, using complementary approaches and technologies. Deletion of the *azu* gene did not affect resistance to antibiotics, copper, or hydrogen peroxide, while nuclear magnetic resonance-based metabolomic analysis revealed that the Δ*azu* strain was moderately impaired in maintaining metabolic homeostasis from the exponential to the stationary growth phase. Phenotype microarray analyses showed that the two strains exhibited largely similar metabolic and resistance profiles, except for bromosuccinic acid utilization, under which the Δ*azu* strain displayed reduced growth. This phenotype was further associated with a reduced ability of the mutant to colonize *Arabidopsis thaliana*, suggesting a role for azurin in maintaining the plant–bacterium association. Overall, these findings provide new insights into the physiological role of azurin in environmental bacteria and suggest its involvement in bacterium–eukaryote interactions, thereby opening new perspectives for biotechnological and biomedical applications.

## 1. Introduction

Azurin, a small water-soluble periplasmic protein composed of 128 amino acids, belongs to the cupredoxin family of copper-containing proteins. It was first identified in the bacterium *Pseudomonas aeruginosa* in 1956 [1] and subsequently purified in 1958 [2]. Structurally, azurin contains a type I copper center that enables efficient single-electron transfer reactions, a feature underlying its involvement in several bacterial redox processes.

Azurin has been extensively investigated in the biomedical field because of its remarkable anticancer properties. Several studies demonstrated that azurin preferentially enters cancer cells over healthy ones [3,4], an ability mainly attributed to the presence of a 28-amino-acid region (p28, Azu 50–77) acting as a transduction domain [5,6]. Once inside the tumor cell, azurin exploits its anticancer activity by complexing with p53 and preventing its degradation [7]. The complex formed by azurin and p53 is imported into the nucleus, where p53 can upregulate the expression of pro-apoptotic genes, such as those encoding for Noxa and Bax proteins [7], leading to the activation of the apoptotic machinery via the release of cytochrome c in the cytosol [5,8,9]. Azurin was also shown to bind the EphB2 tyrosine kinase receptor with high affinity, mimicking the interaction of its natural ligand, ephrinB2; this competitive binding interferes with receptor autophosphorylation, thereby inhibiting tumor cell proliferation and progression [10]. The modulation of cell membrane properties and the inhibition of angiogenesis are additional recognized modes of action involved in azurin and/or p28 antitumoral activity [11].

Besides its anticancer activity, it was also demonstrated that both azurin and the *Neisseria meningitides* azurin-like protein known as Laz [12] inhibit host cell invasion by the AIDS virus HIV-1 and the human parasites *Plasmodium falciparum* and *Toxoplasma gondii* [13,14]. Moreover, the prevention of *Staphylococcus epidermidis* biofilm formation was more recently attributed to cold-azurin, an effective antibiofilm protein produced by an Antarctic marine *Pseudomonas* isolate [15]. These phenomena suggest that azurin-like proteins may be employed as a competitive exclusion strategy against competing microbes (and cancer) attempting to thrive in the same host.

Despite the large amount of data on the antitumoral activity of azurin, little is known about its physiological role in bacteria. Azurin has been functionally and structurally characterized mainly in *P. aeruginosa* [16,17,18,19,20,21], where it has been suggested to be involved in protection against oxidative stress, single-electron transfer during anaerobic respiration, deamination of primary amines, and denitrification [22,23]. In addition to its canonical redox functions, recent evidence suggests that azurin may contribute to copper homeostasis. In *P. aeruginosa*, secretion of azurin through the H2-type VI secretion system was shown to facilitate Cu^2+^ acquisition, thereby conferring a competitive growth advantage under copper-limiting conditions [24]. Further investigations are required to elucidate the precise mechanisms governing azurin localization and secretion, as well as its broader implications in bacterial physiology.

The role of azurin in non-pathogenic pseudomonads [25] and in other taxonomic groups remains poorly explored. A recent study focusing on the phylogenetic distribution of the azurin coding gene (*azu*) revealed that it was absent in all the archaeal and eukaryotic genomes analyzed, whereas it was detected in prokaryotic Phyla other than *Proteobacteria*, such as *Bacteroidota*, *Verrucomicrobia*, and *Chloroflexi* [26]. This comprehensive analysis suggested that, once it appeared in the bacterial domain, the *azu* gene was lost in several bacterial phyla and/or anciently horizontally transferred, even though vertical inheritance appeared to be the major force driving the transmission of this gene [26].

In the present study, we investigated the physiological role of azurin in *Pseudomonas* sp. OHS18, an endophytic strain isolated from the internal tissues of the medicinal plant *Origanum heracleoticum* L. [27]. *Pseudomonas* sp. OHS18 colony is smooth, round-shaped, and convex, with entire margins and an intense orange color. It exhibited a weak antagonistic effect against both endophytic bacterial strains isolated from different plant compartments of *O. heracleoticum* [28] and opportunistic human pathogens belonging to the *Burkholderia cepacia* complex [29]. The strain was isolated only from the stem compartment, suggesting a strong adaptation of the endophyte to its tissue of origin [27,28]. However, the mechanisms underlying the interaction between the endophyte and the plant endosphere were not fully untangled [28,30]. The analysis of the genome of *Pseudomonas* sp. OHS18 revealed the presence of the *azu* gene. To unveil the physiological role that azurin might play in this bacterium, we performed a phenotypic characterization of a mutant (∆*azu*) carrying a deletion of the *azu* gene using a combination of different approaches and technologies. A deeper understanding of the processes mediated by this protein could clarify its role in bacterium–eukaryote interactions and open new avenues for biotechnological and biomedical applications.

## 2. Materials and Methods

### 2.1. Genome Sequencing and Analysis

*Pseudomonas* sp. OHS18 was retrieved from its glycerol (20%) stock and let grow on Tryptic Soy Agar (TSA, Oxoid, Thermo Fisher Scientific, Basingstoke, UK) for 48 h at 30 °C. A single colony of the strain was then inoculated in 10 mL of Tryptic Soy Broth (TSB, Biolife, Milan, Italy) in a 50 mL tube and incubated at 30 °C overnight under shaking (130 rpm). Bacterial cells were collected by centrifugation (15,500× *g* for 4 min) and the cell pellet was subjected to DNA extraction employing the PowerLyzer PowerSoil DNA Isolation Kit (MO BIO Laboratories, Inc., Carlsbad, CA, USA), following the manufacturer’s protocol with some modifications: after resuspending the cells in the PowerSoil Bead Solution, lysozyme was added (1 mg/mL) and the suspension was incubated for 1 h at 37 °C. Then, PowerSoil Solution C1 and proteinase K (0.5 mg/mL) were added to the sample, which was further incubated at 55 °C for 2 h before proceeding with the DNA purification steps.

Nanopore sequencing was performed following the native barcoding genomic DNA protocol provided by Oxford Nanopore Technologies (ONT, Oxford, UK) (v. NBE_9065_v109_revY_14Aug2019). gDNA was repaired and end-prepped using the NEBNext Companion Module for Oxford Nanopore Technologies Ligation Sequencing (E7180S, New England Biolabs, NEB, Ipswich, MA, USA). After purification with Agencourt AMPure XP beads (Beckman Coulter, Brea, CA, USA) on a magnetic separator, the concentration of the DNA sample was obtained using the Qubit dsDNA HS Assay Kit together with a Qubit 4 Fluorometer (ThermoFisher Scientific, Basingstoke, UK). The gDNA was sequenced with 11 other non-related gDNA samples. Thus, 500 ng of the end-prepped DNA samples were barcoded using Native Barcoding Expansion 13–24 (EXP-NBD114, ONT) and NEB Blunt/TA Ligase Master Mix (M0367, New England Biolabs). Equimolar amounts of barcoded DNA samples were pooled to have a total of 700 ng and were subjected to the adapter ligation. During the clean-up step, the library was enriched with >3 kb-long fragments, using the Long Fragment Buffer included in the Ligation Sequencing Kit (SQK-LSK109, ONT). DNA library was immediately sequenced: R9.4.1 Flow Cell (FLO-MIN106D, ONT) was primed with a Flow Cell Priming Kit (EXP-FLP002, ONT). The library was loaded following the instructions provided by the protocol, and sequencing was performed using a MinION MK1B platform (ONT) and the MinKNOW software v.21.10.4 for 72 h. Guppy v.4.3.4 software was used for the basecalling and demultiplexing steps.

De novo assembly was achieved using Canu assembler software v.2.1.1 [31], and the quality of contigs was assessed by QUAST v.5.0.2 [32]. The Average Nucleotide Identity (ANI) analysis was performed using FastANI v.1.3, with default options [33]. The available and complete genomic sequences of *Pseudomonas* strains were downloaded from the NCBI “assembly” database and used as reference input for the ANI analysis. Genome completeness and quality were assessed using BUSCO v5.8.0 [34] against the Pseudomonadales lineage dataset (8 January 2024 pseudomonadales_odb10; 782 Benchmarking Universal Single-Copy Orthologs from 159 reference genomes). Gene prediction was performed with Prodigal [35]. All these functions were performed in a Galaxy environment (https://usegalaxy.eu, accessed on 3 May 2022, and on 7 May 2026 for BUSCO analysis). The complete genome sequence is available in GenBank under the accession number CP182111.

The genome sequence of *Pseudomonas* sp. OHS18 was then uploaded into the Type (Strain) Genome Server (TYGS), a free bioinformatics platform (https://tygs.dsmz.de, accessed on 30 January 2024), for a whole-genome-based taxonomic analysis [36,37]. The results were provided by the TYGS server on 23 March 2024. The assembled genome sequence was annotated using the NCBI Prokaryotic Genome Annotation Pipeline (PGAP) v.6.4 (https://www.ncbi.nlm.nih.gov/genome/annotation_prok/, accessed on 17 February 2025). The antiSMASH v.7.0.1 webserver (https://antismash.secondarymetabolites.org/#!/start, accessed on 18 February 2025) was used for the identification of biosynthetic gene clusters (BGCs).

### 2.2. Deletion of the azu Gene

In-frame deletion of the *azu* gene in *Pseudomonas* sp. OHS18 was performed using a previously described procedure [38] with a few modifications. Two DNA fragments of approximately 500 bp encompassing the upstream and downstream regions of the *Pseudomonas* sp. OHS18 *azu* gene was amplified by PCR, directionally cloned into the sequencing vector pBluescript II (pBS; Stratagene, La Jolla, CA, USA), and verified by DNA sequencing. Primers and restriction enzymes used for PCR and cloning are described in Supplementary Table S1. Then, the DNA fragment encompassing the upstream and downstream regions was excised from pBS and sub-cloned into the *sacB*-based suicide vector pDM4 [39], yielding the deletion mutagenesis construct pDM4Δ*azu*_OHS18_. This construct was transferred from the conjugative *Escherichia coli* strain S17.1λ*pir* [40] into *Pseudomonas* sp. OHS18 by conjugation. Transconjugants, in which the plasmid integrated into the chromosome via homologous recombination, were selected on lysogeny broth (LB) agar plates containing 375 μg/mL chloramphenicol to select plasmid-carrying *Pseudomonas* sp. OHS18 cells, and 25 μg/mL ampicillin, to counterselect *E. coli* cells. A randomly selected transconjugant was cultured in LB until the late-exponential growth phase and then isolated on LB agar plates containing 10% sucrose, which is toxic to cells carrying the *sacB* gene present in the pDM4 backbone [39]. The loss of the plasmid via homologous recombination, which can result in either reversion to the wild-type genotype or in the generation of the deletion mutant, was verified by streaking colonies on LB agar plates supplemented or not with 300 μg/mL chloramphenicol. Chloramphenicol-sensitive clones were screened by colony PCR with the primers *azu* OHS18_UP_FW and *azu* OHS18_UP_RV (Supplementary Table S1) to identify deletion mutants. The absence of the *azu* gene in the deletion mutants (∆*azu*) was further verified by colony PCR with primers internal to the *azu* gene (Supplementary Table S1).

### 2.3. Resistance to Antibiotics, Copper, and Hydrogen Peroxide

The susceptibility of the wild-type and ∆*azu* strains to various compounds, including antibiotics, copper (CuCl_2_), and hydrogen peroxide (H_2_O_2_), was assessed using the broth microdilution method in 96-well plates. Stock solutions of each compound were prepared at a concentration four times higher than the highest tested concentration. 50 µL of Mueller–Hinton (MH) broth was dispensed into the wells of three consecutive rows of the microplate for each compound. Then, 50 µL of the stock solution was added to the first well of each row, achieving an initial 1:2 dilution. Serial twofold dilutions were performed across the wells by transferring 50 µL from one well to the next one, discarding 50 µL from the final well to maintain a consistent volume. Table 1 summarizes the concentrations employed for each compound.

A bacterial suspension was prepared in MH broth (approximately 10^6^ CFU/mL), and 50 µL was added to each well, reaching a final volume of 100 µL per well. Three wells containing only MH broth and the bacterial suspension served as a growth control, while three wells containing only MH broth were used as a blank control. Plates were incubated at 30 °C for 48 h, and OD_600_ was measured using a microplate reader (Infinite 200 PRO multimode reader, Tecan, Männedorf, Switzerland). The mean blank OD value was subtracted from all readings, and the mean OD of triplicate wells was calculated for each dilution and the growth control. The minimum inhibitory concentration (MIC) was defined as the lowest concentration at which bacterial growth was inhibited by ≥80% compared to the growth control.

Growth under copper-limiting conditions was tested in the presence of a Cu^2+^ chelator. Overnight cultures of the wild-type and Δ*azu* strains were diluted to an initial OD_600_ of 0.01 in TSB and TSB supplemented with 0.25 mM EDTA, either with or without 0.1 mM CuSO_4_, following the experimental approach described by Han et al. [24]. Growth was monitored in 96-well microplates using the Infinite 200 PRO multimode reader (Tecan, Männedorf, Switzerland) by measuring OD_600_ every hour for 36 h at 30 °C. The experiment was performed in triplicate. Growth curve parameters were calculated using the growthcurver library in R v.4.4.3 [41] and compared between the wild-type and Δ*azu* strains for each growth condition using the Wilcoxon–Mann–Whitney test (*p* < 0.05). Growth curves visualization was performed using the ggplot2 library in R [42].

### 2.4. Nuclear Magnetic Resonance (NMR) Spectroscopy

Sample preparation for ^1^H NMR-based metabolomics was performed according to procedures developed at CERM for the analysis of prokaryotic cells [43,44,45]. A single colony of *Pseudomonas* sp. OHS18 wild-type or ∆*azu* strains were inoculated in 10 mL of sterile TSB. Four precultures were prepared for each strain and were incubated overnight at 30 °C with shaking. Each of the obtained precultures was used to start a new bacterial culture with an initial OD_600_ = 0.1, in a final volume of 200 mL of TSB. The flasks were incubated at 30 °C with shaking. Cell growth was monitored by measuring the OD_600_ every hour. At each selected time point, namely early and late exponential growth (2.5 h and 4 h) and early and late stationary phase (8 h and 24 h), one of the four cultures was processed. In particular, 1 mL of the medium was filtered (Filtropur 0.2 μm, SARSTED AG & Co. KG, Nümbrecht, Germany) to remove bacterial cells and kept at −20 °C. The remaining culture (199 mL) was pelleted by centrifugation for 10 min at 11,000 rpm at 4 °C and stored at −20 °C. 1 mL of fresh TSB was also maintained at −20 °C (blank control). The whole experiment was performed in triplicate. Once all samples were collected, pelleted cells were thawed, resuspended in 500 µL of PBS, and sonicated for 20 min (1 s of activity and 9 s of rest, 292.5 W, 13 mm tip), with contemporary cooling on ice. The cytosolic fractions containing the soluble metabolites were obtained upon centrifugation at 17,000× *g* for 1 h at 4 °C and stored at −80 °C until NMR analysis.

For NMR sample preparation, all samples were thawed at room temperature; for cell lysates, 55 μL of ^2^H_2_O was added to 495 μL of each sample. For culture media (filtered spent medium and the blank control), 60 μL of sodium phosphate buffer (70 mM Na_2_HPO_4_; 20% *v*/*v* ^2^H_2_O; 4.6 mM TMSP, pH 7.4) was added to 540 μL of each medium. Mixtures were homogenized by vortexing for 30 s and transferred into 5 mm NMR tubes (Bruker BioSpin srl, Billerica, MA, USA).

NMR-based metabolomic analysis was performed according to standard procedures [46]. ^1^H NMR spectra were recorded with a Bruker 600 MHz spectrometer (Bruker BioSpin) optimized for metabolomic analysis, operating at 600.13 MHz proton Larmor frequency and equipped with a 5 mm PATXI ^1^H–^13^C–^15^N and ^2^H-decoupling probe including a *z*-axis gradient coil, an automatic tuning-matching (ATM), and an automatic refrigerated sample changer (SampleJet, Bruker BioSpin). A BTO 2000 thermocouple served for temperature stabilization at a level of approximately 0.1 K of the sample. To equilibrate the temperature at 300 K, samples were kept for at least 5 min inside the NMR probe head before measurements.

All the samples were analyzed using the Carr–Purcell–Meiboom–Gill (CPMG) sequence using a one-dimensional (1D) spin-echo sequence with water presaturation. 73,728 data points, a spectral width of 12 019 Hz, and a relaxation delay of 4 s were used. 128 and 64 scans were used for cell lysates and growth media, respectively.

All the raw data were multiplied by a 0.3 Hz exponential line broadening before applying the Fourier transform. Transformed spectra were automatically corrected for phase and baseline distortions. The spectra were calibrated at the doublet of Ala at 1.49 ppm using TopSpin 3.6 (Bruker Biospin srl).

The metabolites whose peaks in the spectra were well resolved were assigned, and their levels were analyzed. The assignment was performed using Chenomx software v.8.3. The relative quantification of the NMR signals was performed using an R script developed in-house. In total, 34 and 47 metabolites were identified and quantified in the cell lysate and in the growth medium spectra, respectively (Supplementary Table S2). In the growth medium, the metabolites were divided into two different classes, i.e., those that are “taken up” from the medium (23 metabolites) and those that are “released” into the medium (24 metabolites). The definition of “taken-up” or “released” has to do with the concentration balance with respect to the media composition at time zero (blank): a taken-up metabolite is one whose concentration in the growth medium at a given time is lower than at time zero; a released metabolite in one whose concentration in the growth medium is higher than at time zero (or which was not present at time zero). Metabolite intensities were normalized to the total area, calculated with the exclusion of the water peak.

On the assumption that metabolite concentrations are not normally distributed, the nonparametric pairwise Wilcoxon–Mann–Whitney test was used for the determination of the meaningful metabolites in the comparison between wild-type and ∆*azu* at each of the four selected time points.

### 2.5. Phenotype Microarray

Phenotype microarray analysis was performed using the Biolog GEN III MicroPlates (Catalog No.1030, Biolog), containing 71 carbon sources and 23 chemical sensitivity assays. All the reagents and materials used were provided by Biolog, Inc. (Hayward, CA, USA). For each strain (wild type and ∆*azu*), a bacterial suspension was prepared by resuspending a few bacterial colonies in the IF-A inoculation fluids (Catalogue No.72401, Biolog), in triplicate. Suspensions of 98% transmittance were obtained, and 100 μL were dispensed into each well of a Biolog GEN III MicroPlate. The microplates were incubated in the Omnilog reader (Biolog) at 30 °C for 4 days. Color development was recorded by the automated Omnilog System every 15 min until reaching the plateau phase. Results obtained were analyzed through the DuctApe software suite (version 0.18.2) [47]. The DuctApe software allowed us to obtain a standardized Activity Value (AV) for each compound, representing the metabolic activity of the strains in the presence of each compound. Some of the graphical visualizations (maximum height, average Y-value, and initial rate) were obtained using the Biolog Data Analysis software (v 1.7). The heatmap construction was carried out in the R software, using the pheatmap library [48].

### 2.6. Bacterial Plant Colonization

Plantlets of *Arabidopsis thaliana* (ecotype Col-0) were grown in vitro. Seeds were surface-sterilized and germinated on solid ½ Murashige and Skoog (MS) medium supplemented with 1% (*w*/*v*) sucrose and 10% agar (24 °C, 16 h light/8 h dark photoperiod). After germination, 5-day-old plantlets of identical size were transferred to large square Petri dishes with a maximum of four plants per plate and allowed to grow vertically for one or two days more. Plantlets were monitored daily to detect any microbial contamination.

Wild-type and ∆*azu* strains were streaked onto TSA plates and incubated at 30 °C for 48 h. A single colony from each strain was used to inoculate 10 mL of TSB, which was incubated overnight at 30 °C with shaking at 130 rpm. On the day of inoculation, bacterial cultures were diluted 1:10 directly in a spectrophotometric cuvette, and optical density at 600 nm (OD_600_) was measured. Based on this measurement, the appropriate volume of culture was calculated to obtain a final OD_600_ of 1.0 in a total volume of 5 mL of TSB. Bacterial suspensions were centrifuged for 15 min at 4500 rpm; the supernatant was discarded, and the pellet was resuspended in 5 mL of sterile physiological saline solution (0.9% NaCl). OD_600_ was measured again, and bacterial suspensions were diluted in physiological saline solution to obtain a final OD_600_ of 0.01 in a total volume of 5 mL. This suspension was used for plant inoculation.

Using a sterile micropipette, 50 µL of the bacterial suspension (OD_600_ = 0.01) was carefully dispensed onto the agar medium in close proximity to the root system or to the basal portion of the hypocotyl immersed in the medium. Control plants were mock-inoculated with 50 µL of sterile physiological saline solution. After inoculation, plantlets were returned to the growth chamber and maintained under standard growth conditions for at least two weeks. At least three biological replicates were prepared for each condition. To quantify the bacterial inoculum, serial dilutions (10^−2^ and 10^−4^) of the OD_600_ = 0.01 suspension were prepared in physiological saline solution. Aliquots of 100 µL of the undiluted suspension, 10^−2^, and 10^−4^ dilutions were plated on TSA and incubated at 30 °C for 24 h. Colony-forming units (CFU) were counted the following day.

Endophytic bacteria were isolated from in vitro–grown *A. thaliana* plantlets. Residual culture medium was removed, and plantlets were surface-sterilized by immersion in 1% (*v*/*v*) sodium hypochlorite (NaClO) for up to 2.5 min, followed by three rinses with sterile distilled water. To verify surface sterilization, 100 µL of the final rinse was plated on TSA and incubated at 30 °C for 24 h. Surface-sterilized tissues were briefly air-dried under sterile conditions and separated into leaves and stems. For each replicate, tissues were pooled by type, and 100 mg (fresh weight) was homogenized in 500 µL of sterile physiological saline solution (0.9% NaCl). Homogenates were centrifuged briefly at low speed to remove plant debris, and supernatants were serially diluted and plated on TSA. Plates were incubated at 30 °C for 24 h, after which colony-forming units (CFU g^−1^ FW) were counted. CFU values were derived from 3 independent biological replicates of 5 pooled plants per replicate. Wild-type and Δ*azu* microbial loads were compared separately within each tissue (leaves and roots). Data were analyzed after a log-10 transformation, using Welch’s unpaired two-tailed *t*-test.

## 3. Results and Discussion

### 3.1. Genome Analysis Revealed the Presence of the azu Gene in the Genome of Pseudomonas *sp.* OHS18

Whole-genome sequencing was performed to investigate the presence of the *azu* gene and other genomic features in *Pseudomonas* sp. OHS18. The genome assembly consists of a single contig of 5,228,754 bp and a GC content of 63.35% (Table 2). Benchmarking Universal Single-Copy Orthologs (BUSCO) analysis revealed high genome completeness with 97.8% of complete BUSCOs (765/782), of which 96.4% were single-copy (754/782) and 1.4% were duplicated (11/782). Fragmented BUSCOs were absent, while 2.2% of BUSCOs were missing (17/782), overall indicating a high-quality genome assembly.

Average Nucleotide Identity (ANI) and digital DNA–DNA hybridization (dDDH) analyses were also performed. The genome sequence of *Pseudomonas* sp. OHS18 showed an ANI value < 95% with respect to all the tested genomes (the closest strain was *Pseudomonas argentinensis* LMG 22563, NCBI RefSeq assembly GCF_900113905.1, with an ANI value of 92.39%); moreover, the genome sequence did not match any species found in the TYGS database, showing a maximum dDDH (formula *d4*) value of 44.4% with *Pseudomonas argentinensis* LMG 22563. Altogether, these results suggest that OHS18 may represent a novel *Pseudomonas* species.

Secondary metabolite biosynthetic gene clusters (BGCs) analysis predicted six different BGCs (Table 3); however, only one BGC had a percentage of similarity higher than 50% with clusters available in the database. This BGC is predicted to direct carotenoid biosynthesis, with a 100% similarity to the carotenoid BGC from *Enterobacteriaceae* [49]. Carotenoids are natural pigments, producing light yellow to orange to deep red colors [50]. These pigments have important functions in photosynthesis, nutrition, and protection against photooxidative damage [50]. The presence of this BGC might be responsible for the orange pigmentation of *Pseudomonas* sp. OHS18. Moreover, carotenoids have been associated with protection against oxidative and environmental stress in bacteria [51], suggesting a potential adaptive role in the plant endosphere.

Besides BGCs, the genome of *Pseudomonas* sp. OHS18 strain harbors the *azu* gene; the gene sequence as well as the amino acid sequence of the encoded azurin protein are reported in Supplementary Material (Supplementary Figure S1). Azurin protein from *Pseudomonas* sp. OHS18 showed 66.44% identity and 78.52% similarity with the one from *P. aeruginosa*, as determined by ClustalW alignment (Supplementary Figure S1). To investigate the role of azurin in *Pseudomonas* sp. OHS18, we generated an in-frame deletion [38] of 372 bp in the *azu* gene, corresponding to 83% of the coding sequence. The resulting mutant (Δ*azu*) was compared to the parental strain in several phenotypic assays to assess whether, and to what extent, azurin affects *Pseudomonas* sp. OHS18 physiology. No significant differences in aerobic growth kinetics were observed between the Δ*azu* mutant and the wild-type strain under the tested conditions (Supplementary Figure S2), in accordance with what was previously observed for an azurin-deficient *P. aeruginosa* PAO1 strain [52].

### 3.2. Deletion of the Azurin Coding Gene Does Not Alter Antibiotic Susceptibility, Oxidative Stress Tolerance, or Growth Under Copper-Limiting Conditions

As a first step, we investigated whether *azu* deletion increases susceptibility to specific antibiotics. Antibiotic resistance is one of the major public health challenges of recent decades, turning once-manageable infections into potentially life-threatening conditions. Although *Pseudomonas* sp. OHS18 is an environmental endophyte rather than a clinical pathogen, its antibiotic-response mechanisms may still provide insights into resistance strategies of related opportunistic species, including *P. aeruginosa*. As shown in Table 4, results showed that the deletion of the *azu* gene did not affect the susceptibility of *Pseudomonas* sp. OHS18 to any of the tested antibiotics, since the MIC values were identical to those of the wild-type strain. The highest MIC values were obtained for tetracycline and chloramphenicol, while the strains exhibited the highest sensitivity to kanamycin and ciprofloxacin.

Concerning azurin redox activity, previous studies reported a markedly increased sensitivity to hydrogen peroxide in an azurin-deficient mutant of *P. aeruginosa* PAO1 [52]. It was therefore proposed that the physiological role of azurin involves electron transfer directly related to the cellular response to redox stress [52]. In our experimental setting, however, deletion of the *azu* gene did not affect *Pseudomonas* sp. OHS18 susceptibility to hydrogen peroxide, suggesting that azurin is not directly involved in mediating resistance to this stressor in this strain, or that alternative mechanisms may compensate for its loss.

**Figure 1:**
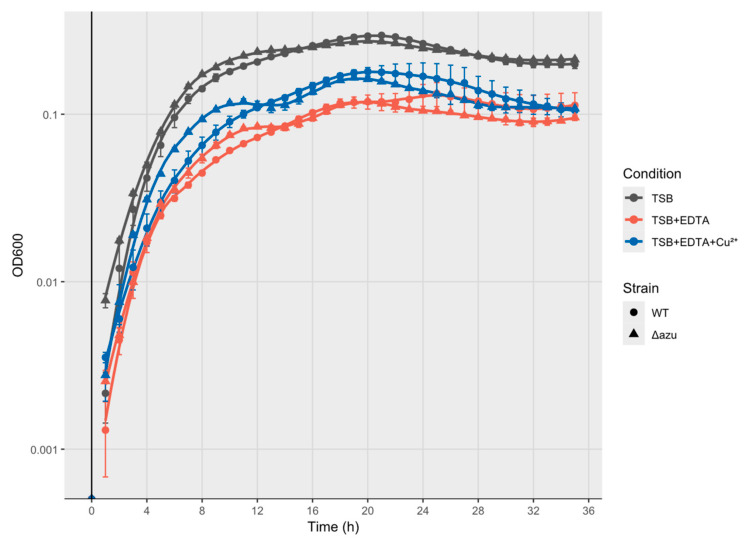
Growth kinetics of wild-type (WT) and Δ*azu* strains in TSB medium under different conditions. Bacteria were grown in TSB alone and TSB supplemented with EDTA (0.25 mM), with or without CuSO_4_ (0.1 mM). Growth was monitored by measuring optical density at 600 nm (OD_600_). Data are reported on a logarithmic scale. WT and Δ*azu* strains are represented by circles and triangles, respectively. Each growth condition is shown in a different color, as indicated in the legend on the right. Error bars represent standard error.

Moreover, although azurin has been implicated in redox processes and metal interactions in other *Pseudomonas* species [53,54], azurin deletion in *Pseudomonas* sp. OHS18 did not influence copper tolerance. It was recently evidenced that under low Cu^2+^ conditions, the H2-type VI secretion system (H2-T6SS) of *P. aeruginosa* is upregulated, leading to the extracellular secretion of azurin. Once secreted, azurin binds environmental Cu^2+^ and delivers it to the outer membrane transporter OprC, facilitating copper uptake [24]. This T6SS-Azu-OprC system represents a novel mechanism for maintaining copper homeostasis and highlights the adaptability of the bacterium to fluctuating metal availability [24]. Orthologous genes related to the T6SS-Azu-OprC system were identified in the genome of *Pseudomonas* sp. OHS18. To further investigate the proposed role of azurin in copper acquisition under copper-limiting conditions, the growth of the wild-type and Δ*azu* strains was monitored in TSB supplemented with 0.25 mM EDTA, either in the absence or presence of 0.1 mM CuSO_4_ (Figure 1).

As expected, EDTA supplementation reduced bacterial growth, whereas the addition of CuSO_4_ partially restored growth in both strains. However, no significant differences were detected between the wild-type and Δ*azu* strains for any of the analyzed growth parameters under either condition (Supplementary Table S3). Further investigations are required to clarify whether azurin from *Pseudomonas* sp. OHS18 plays a role in metal homeostasis under specific environmental conditions or in synergy with other cellular components.

### 3.3. Azurin Is Involved in the Maintenance of Metabolic Homeostasis in Pseudomonas *sp.* OHS18 During the Transition to the Stationary Phase

To further characterize the role of azurin in bacterial cell metabolism, we performed NMR spectroscopy-based metabolic analyses. At four different time points (mid-logarithmic phase, end of logarithmic phase, early stationary phase, and late stationary phase), 1 mL of filtered culture (released and taken up metabolites) and the pelleted cells (intracellular metabolites) were collected, stored at −20 °C, and further processed as described in Materials and Methods. The results are summarized in Supplementary Table S2.

The intracellular level of several metabolites differed significantly between the two strains during bacterial growth (Table 5).

In particular, the intracellular concentration of 3-methyl-2-oxovalerate, a branched-chain keto acid whose levels generally decreased over time, was significantly lower in the Δ*azu* mutant during the late exponential phase. Conversely, glucose-1-phosphate (G1P) and glutamate, both of which progressively accumulated inside the cell, were present at higher concentrations in the Δ*azu* strain during the late exponential phase and early stationary phase, respectively. In the late stationary phase, intracellular choline levels were lower in the mutant strain (Supplementary Figure S3).

Differences were also observed in the extracellular metabolomic profiles (Supplementary Figure S4). During the late exponential phase, the Δ*azu* strain released lower amounts of lysine, whereas the extracellular levels of threonine and hydroxyacetone were increased compared with the wild-type strain. In the late stationary phase, acetate release was higher in the Δ*azu* mutant. In contrast, proline was progressively depleted from the extracellular medium during bacterial growth, indicating cellular uptake. Proline uptake was consistently higher in the Δ*azu* strain at all time points, although the difference was statistically significant only during the late exponential phase.

Analysis of the metabolomic profiles revealed that *azu* deletion induced a metabolic shift affecting both central carbon and nitrogen pathways. To the best of our knowledge, azurin has not previously been associated with these metabolic pathways. Moreover, the presence of a predicted N-terminal signal peptide suggests an extracytoplasmic (periplasmic) localization of the protein, making a direct role in intracellular metabolic reactions unlikely. Instead, given its established role in electron transfer, we hypothesize that azurin indirectly affects the metabolic flexibility required to coordinate carbon and nitrogen fluxes during specific bacterial growth stages. It was previously observed that azurin expression is induced by two distinct growth conditions in *P. aeruginosa* PAO1, namely, anaerobiosis and the transition from the exponential to the stationary phase, suggesting a functional relationship between azurin and the metabolic adaptation to redox imbalance during growth arrest and oxygen limitation [52]. Consistently, most of the metabolic differences between the two strains were observed at the end of the exponential phase. In this context, the absence of azurin may have impaired *Pseudomonas* sp. OHS18’s ability to maintain redox homeostasis during this critical transition, characterized by nutrient limitation, waste accumulation, and the progressive decline in growth rate.

The overall impact of azurin deletion on the metabolism of *Pseudomonas* sp. OHS18 should be interpreted with caution, as direct mechanisms cannot be extrapolated from this analysis. Nevertheless, some of the detected metabolic changes likely reflect the activation of stress-responsive metabolic mechanisms in the Δ*azu* mutant. In particular, a stronger depletion of extracellular proline was detected in the mutant during the late exponential phase, followed by a higher intracellular concentration of glutamate during the early stationary phase. Proline is a known precursor of glutamate, a metabolite connecting energy production and amino acid metabolism, also involved in stress responses to acid, osmotic, and oxidative stresses [55,56,57]. In this context, the increased proline uptake (and the subsequent intracellular glutamate increase) may reflect a more pronounced effort to cope with oxidative and osmotic stress, compared to the wild-type strain.

Choline was detected in both intracellular and extracellular fractions and categorized as a “taken up” metabolite, as its extracellular concentration progressively decreased relative to the initial growth medium, particularly during the late stationary phase. This observation is consistent with previous evidence showing that pseudomonads lack a characterized de novo choline biosynthetic pathway, while transport systems for exogenous choline uptake are widespread within this bacterial group [58]. In bacteria, choline primarily serves as a precursor of glycine betaine, an osmoprotective compound involved in resistance to osmotic, thermal, and oxidative stresses, as well as in intracellular pH homeostasis [58]. The lower intracellular concentration of choline observed in the Δ*azu* strain during the late stationary phase suggests an enhanced conversion of this metabolite into glycine betaine as part of a stress-adaptive response.

Further investigation would be required to establish whether redox imbalance or other indirect consequences of the loss of this periplasmic protein are the underlying mechanisms driving the observed metabolic shifts.

### 3.4. Deletion of the Azurin Coding Gene from Pseudomonas *sp.* OHS18 Was Associated with Reduced Bromosuccinic Acid Utilization in Phenotype Microarray Assays

**Figure 2:**
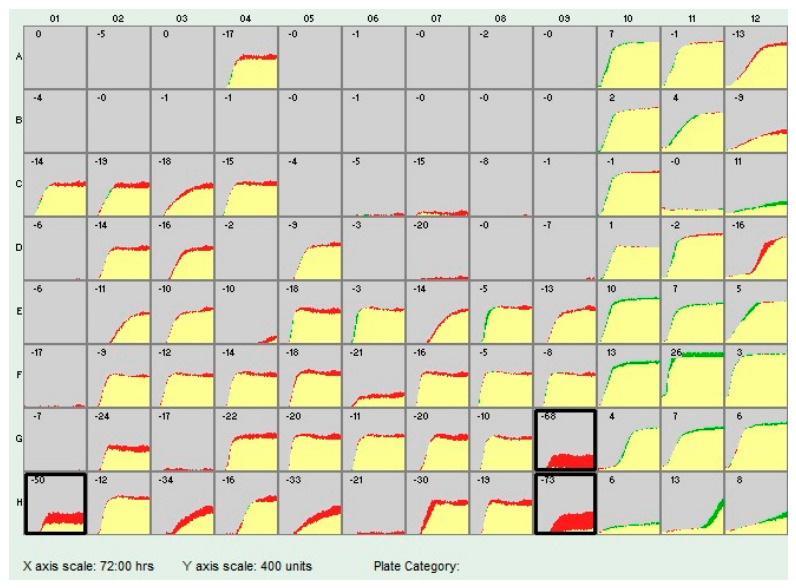
Metabolic activity curves of the wild-type (red) and Δ*azu* (green) strains in the presence of carbon sources (columns 1–9) and inhibitory compounds (columns 10–12) from the GEN III microplate (Biolog). Yellow indicates the overlap between the growth curves of the two strains. Differences between strains are expressed as Biolog Units (BU), representing the magnitude of the difference in metabolic activity for each substrate (values shown in each panel). Positive values indicate higher substrate utilization by the mutant strain relative to the wild type, whereas negative values indicate lower utilization. Values between −49 and +49 BU were considered not significantly different according to the software threshold setting. Highlighted panels (G09, H01, and H09) indicate compounds that were differentially utilized by the two strains.

The ability of the wild-type and Δ*azu* strains to utilize different carbon sources and their susceptibility to chemical stressors were further evaluated using phenotype microarrays. The assay provides a phenotypic fingerprint of the microorganisms, enabling a sensitive screening of both carbon source utilization and resistance to inhibitory compounds. Figure 2, obtained via the Biolog Data Analysis software (v 1.7), depicts the metabolic activity curves of the two strains in each well of the GEN III microplate. The two strains exhibited largely similar metabolic profiles across the 23 sensitivity assays (columns 10–12) and 71 carbon sources (columns 1–9), in agreement with the NMR results. Nevertheless, in the presence of bromosuccinic acid (G09), formic acid (H09), and Tween 40 (H01), the Δ*azu* strain showed absent or reduced metabolism. These differences were further supported by analyses of individual kinetic parameters using the Biolog Data Analysis software (Supplementary Figures S5–S7).

**Figure 3:**
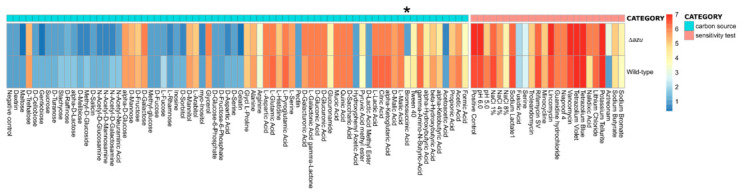
Heatmap representation of the substrate utilization and sensitivity profiles expressed as Activity Values (AV). Rows represent ∆*azu* and wild-type strains, while columns represent different compounds in the GEN III plate. The color notations on the right side of the plot represent the category of each compound (carbon source or sensitivity test). Statistically significant differences (Mann–Whitney test, *p* < 0.05) are marked with an asterisk.

To obtain a more comprehensive and quantitative overview of metabolic and sensitivity profiles, Activity Values (AVs) for each compound in the GEN III plate were calculated using the Ductape software [47]. Mean values from three replicates were then used to generate a heatmap (Figure 3), which highlighted a marked difference between the two strains in the utilization of bromosuccinic acid. The non-parametric Mann–Whitney test confirmed that the difference in AVs between the wild-type and Δ*azu* strains in the presence of bromosuccinic acid was statistically significant (*p* = 0.04285).

Bromosuccinic acid (C_4_H_5_BrO_4_) is succinic acid substituted at position 2 by a bromine atom. It is a dicarboxylic acid, a 2-bromocarboxylic acid, and a conjugate acid of bromosuccinate. In the literature, impaired bromosuccinic acid utilization has been associated with several bacterial phenotypes. In the study by Shi et al. (2007), bromosuccinic acid metabolism was linked with protein secretion and energy conservation systems. Specifically, the study examined the twin-arginine translocation (TAT) system, which plays a crucial role in energy metabolism and the respiratory chain and is responsible for transporting folded proteins and enzyme complexes into the periplasm [59]. Notably, a mutant strain lacking the *tatC* gene completely lost its ability to metabolize bromosuccinic acid, linking utilization of this substrate to the functionality of the TAT secretion system [59]. Additionally, (bromo)succinic acid is among the organic acids found in plant tissues and root exudates, alongside malate and citrate [60]. Bacteria rely on various signal transduction systems to sense these compounds and generate adaptive responses, such as chemotaxis towards plant roots and eventually plant tissue colonization [61]. The study by Oksinska et al. (2011) compared the nutrient utilization profiles of different *Pseudomonas* strains exhibiting varying colonization abilities in wheat plants [62]. Bromosuccinic acid was among the compounds preferentially utilized by the most effective plant colonizers. Similarly, the ability to metabolize bromosuccinic acid was related to a higher rhizosphere colonization ability of phenazine-producing *Pseudomonas* species [63]. Additionally, bromosuccinic acid was one of the carbon sources utilized by some *Pseudomonas* species isolated from the rhizosphere of wheat, which were able to produce 2,4-diacetylphloroglucinol (DAPG) [64], a strong biocontrol agent against phytopathogens [65]. The tricarboxylic acid cycle-responsive chemoreceptors identified in *Pseudomonas* spp. include McpK, which specifically responds to α-ketoglutarate, and PA2652, which is a malate-specific receptor [66,67]. Notably, it was demonstrated that the *P. putida* PA2652 receptor could also bind various C2-substituted C4-dicarboxylic acids, including D,L-bromosuccinic acid [66]. Quantitative chemotaxis tests on a PA2652-deficient mutant revealed a dramatic reduction in chemotaxis for bromosuccinic acid [66]. However, plant colonization experiments did not reveal significant differences in attraction between the wild-type and mutant strains [66].

### 3.5. Azurin Production Influences Both Host Growth Responses and *Pseudomonas* *sp.* OHS18 Persistence During Plant Colonization

Considering the endophytic nature of *Pseudomonas* sp. OHS18, we decided to evaluate whether the deletion of the *azu* gene could also influence the strain’s ability to colonize plant tissues. A preliminary genomic analysis of bacterial species harboring the *azu* gene revealed that these species typically establish interactions (often pathogenic ones) with eukaryotic hosts, including plants and animals [26]. To investigate whether azurin contributes to bacterium–host interactions in this endophytic strain, we performed plant colonization experiments using the wild-type and the mutant strains in the model plant *A. thaliana*.

**Figure 4:**
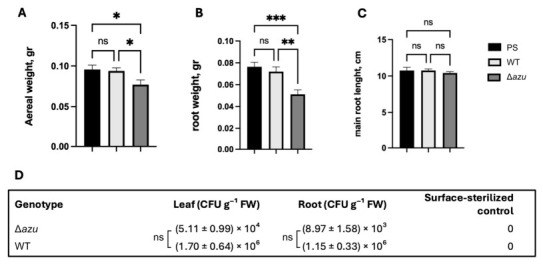
Growth parameters and endophytic bacterial colonization in *A. thaliana* Col0 plants inoculated with *Pseudomonas* sp. OHS18 wild-type (WT) and ∆*azu*. (**A**) Aerial fresh weight, (**B**) root fresh weight, and (**C**) primary root length of plants grown under the indicated conditions. Bars represent SEM. Black bars indicate mock-treated controls (PS), white bars indicate WT-inoculated plants, and gray bars indicate Δ*azu*-inoculated plants. Statistical significance was determined by one-way ANOVA followed by Tukey post hoc multiple comparison tests (**A**–**C**); ns, not significant; * *p* < 0.05; ** *p* < 0.01; *** *p* < 0.001. (**D**) Endophytic bacterial populations in leaves and roots of WT- and Δ*azu*-inoculated plants, expressed as colony-forming units per gram of fresh weight (CFU g^−1^ FW). Values are reported as mean ± SEM. Unpaired two-tailed tests were used to compare WT and Δazu colonization levels within each tissue, using log10-transformed CFU g^−1^ FW values; ns, not significant. Surface-sterilized controls showed no detectable bacterial growth, confirming the effectiveness of surface sterilization.

Inoculation assays using *A. thaliana* and *Pseudomonas* sp. OHS18 wild-type and ∆*azu* strains suggested that azurin production influences both host growth responses and bacterial persistence during the interaction. Plants grown in association with the azurin-producing wild-type strain developed similarly to mock-treated controls, showing no measurable changes in overall growth, whereas plants exposed to the ∆*azu* mutant consistently displayed a slight but reproducible decrease in size (Figure 4A–C). Analysis of bacterial populations recovered from plant tissues showed that the wild-type strain reached higher colonization levels than the Δ*azu* mutant, suggesting that azurin contributes to bacterial persistence in planta (Figure 4D). However, these differences were not statistically significant; thus, data on bacterial colonization should be interpreted as a trend and not as clear evidence of differential endophytic colonization. Interestingly, while there was no significant difference in the endophytic bacterial populations between wild-type and Δ*azu* strains, Δ*azu*-inoculated plants displayed different growth and developmental characteristics compared to wild-type-inoculated plants. This suggests that the observed plant phenotype may not be solely dependent on the final recoverable bacterial load, but may reflect qualitative differences in bacterial activity, plant–bacterium signaling, timing of colonization, tissue localization or the ability of the WT strain to modulate plant physiological pathways.

Plant-associated *Pseudomonas* species are known to establish interactions ranging from beneficial to neutral, often relying on the modulation of host defense and stress responses rather than overt pathogenicity [68,69]. In this context, given its redox-active properties, azurin may contribute to the modulation of the redox environment during colonization, potentially affecting ROS-associated plant responses, thereby limiting excessive activation of reactive oxygen species-mediated defenses, which are known to impose substantial physiological costs on plant growth [70,71]. The trend observed for the azurin-producing OHS18 strain to persist at higher levels without negatively affecting plant development supports a model in which azurin contributes to interaction stability, likely by modulating host physiological or defense responses rather than promoting growth or virulence.

In conclusion, these results suggest that azurin might impact plant growth and development, while its contribution to endophytic colonization needs further validation using complementary localization approaches. Transcriptomic analysis of both bacterial and plant tissues during colonization, confocal microscopy to visualize bacterial localization, and chemical analysis of root exudates to identify relevant metabolites would be necessary to clarify the molecular basis of azurin’s role in plant colonization.

## 4. Conclusions

Beyond its well-established antitumoral activity, the physiological role of azurin in *Pseudomonas* species and in environmental contexts remains poorly understood. In this work, we investigated the physiological function of this protein in the endophytic bacterium *Pseudomonas* sp. OHS18 through the generation of an azurin-deficient mutant. Although resistance assays did not fully confirm a direct role for azurin in stress tolerance, NMR-based metabolomic profiling suggested its involvement in redox balance, as the Δ*azu* strain exhibited a moderate metabolic shift during the transition from exponential to stationary phase. More notably, phenotype microarray analyses, together with the in vivo colonization experiments in *A. thaliana*, suggested a potential role for azurin in plant–bacteria interactions. However, the link between the in vitro metabolic defect and the in vivo colonization phenotype remains speculative and warrants further investigation. Additional validation experiments using defined media with bromosuccinic acid as the sole carbon source will be important to further confirm this observation. Additionally, it would be valuable to further investigate the colonization ability of the mutant strain in the presence of other members of the plant endophytic microbiome. Indeed, given that azurin-like proteins have also been classified as a bacteriocin [72], azurin production may contribute to successful plant colonization by inhibiting the entry of competing strains within the plant endosphere, thereby supporting niche establishment and persistence.

## Figures and Tables

**Table 1:** Antibiotics, copper, and hydrogen peroxide concentrations used to test the bacterium susceptibility in the microdilution test.

Compound	Concentrations					
Streptomycin (μg/mL)	50	25	12.5	6.25	3.125	
Tetracycline (μg/mL)	50	25	12.5	6.25	3.125	
Kanamycin (μg/mL)	25	12.5	6.25	3.12	1.563	
Chloramphenicol (μg/mL)	200	100	50	25	12.5	
Ciprofloxacin (μg/mL)	2.5	1.25	0.625	0.313	0.156	
Rifampicin (μg/mL)	20	10	5	2.5	1.25	
CuCl_2_ (mM)	80	40	20	10	5	2.5
H_2_O_2_ (%)	0.01	0.005	0.0025	0.00125	0.000625	0.0003125

**Table 2:** General features of the genome of *Pseudomonas* sp. OHS18.

Attributes	Value
Genome size	5,228,754
Contigs	1
G + C %	63.35
Total genes	4985
CDS	4905
rRNA	16
tRNA	64
ncRNA	4

**Table 3:** Biosynthetic gene clusters (BGCs) identified using AntiSMASH. For each BGC, the nucleotide (nt) position within the genome is reported, together with the BGC type and the most similar known cluster.

BGC Type ^1^	From (nt)	To (nt)	Most Similar Known Cluster	Similarity
Betalactone	132,440	163,211	-	-
NRPS, NRPS-like, NRP-metallophore	173,442	240,280	cupriachelin (NRP siderophore)	35%
RiPP-like, aryopolyene, resorcinol	1,804,288	1,864,336	APE Vf	40%
Redox cofactor	2,691,947	2,713,998	lankacidin C (NRP + Polyketide)	13%
NAGGN	4,618,887	4,634,033	O-antigen (Saccharide)	21%
Terpene	5,019,082	5,042,733	Carotenoid (Terpene)	100%

^1^ Abbreviations: NRP (Nonribosomal Peptide); RiPP (Ribosomally synthesized and post-translationally modified peptides); NAGGN (N-Acetylglutaminylglutamine amide); APE (arylpolyene).

**Table 4:** Minimal inhibitory concentration for each of the tested molecules.

Tested Molecules		Wild-Type	Δ*azu*
Antibiotics	Tetracycline (µg/mL)	25	25
	Streptomycin (µg/mL)	3.125	3.125
	Kanamycin (µg/mL)	1.563	1.563
	Chloramphenicol (µg/mL)	25	25
	Ciprofloxacin (µg/mL)	0.156	0.156
	Rifampicin (µg/mL)	2.5	2.5
Redox stress	H_2_O_2_ (%)	0.00125	0.00125
Copper sensitivity	CuCl_2_ (mM)	20	20

**Table 5:** NMR metabolic signature of *Pseudomonas* sp. OHS18 Δ*azu* mutant during different growth phases, compared to the wild-type strain. Arrows indicate metabolites whose relative abundance was significantly higher (“↑”) or lower (“↓”) in the Δ*azu* mutant than in the wild-type strain. Only statistically significant differences are here reported (Wilcoxon–Mann–Whitney test, *p* < 0.05).

Growth Phase	Compound	Concentration Differences	Δ*azu* Metabolic Changes
Late exponential	Glucose-1-phosphate	↑ Intracellular	Higher intracellular accumulation
	3-Methyl-2-oxovalerate	↓ Intracellular	Lower intracellular accumulation
	Threonine	↑ Extracellular	Greater release
	Hydroxyacetone	↑ Extracellular	Greater release
	Lysine	↓ Extracellular	Reduced release
	Proline	↓ Extracellular	Greater uptake
Early stationary	Glutamate	↑ Intracellular	Higher intracellular accumulation
Late stationary	Choline	↓ Intracellular	Lower intracellular accumulation/lower uptake
	Acetate	↑ Extracellular	Greater release

## Data Availability

The raw data supporting the conclusions of this article will be made available by the authors on request.

## Supplementary Materials

The following supporting information can be downloaded at: https://www.mdpi.com/article/10.3390/microorganisms14071499/s1, Table S1: Primers used in this study; Table S2: List of metabolites identified by NMR-based metabolomic analysis; Figure S1: *Pseudomonas* sp. OHS18 azurin coding gene, its amino acid sequence, and its alignment with that of *P. aeruginosa* PA01; Figure S2: Growth kinetics of wild-type (WT) and Δ*azu* strains in Tryptic Soy Broth (TSB); Table S3: Growth curve parameters of wild-type (WT) and Δ*azu* strains under different culture conditions (TSB + EDTA 0.25 mM, and TSB + EDTA + CuSO_4_); Figure S3: Boxplots showing compounds differentially detected in cell lysates; Figure S4: Boxplots showing compounds differentially detected in the spent media; Figure S5: Plot showing the average Y-values of the growth curves for each compound in the Biolog GenIII microplate; Figure S6: Plot showing the average initial rates values (Biolog Units per hour) of the growth curves for each compound in the Biolog GenIII microplate; Figure S7: Plot showing the average maximum height values (Biolog Units) of the growth curves for each compound in the Biolog GenIII microplate.
