# Supplementary material for: Exploring the Role of Azurin from the Endophytic Bacterium Pseudomonas sp. OHS18 Through the Phenotypic Characterization of a Δazu Mutant
Source: Microorganisms. 2026 Jul 9;14(7):1499. doi: 10.3390/microorganisms14071499 (PMC13413535; doi:10.3390/microorganisms14071499)
Supplement: Supplementary file 1 [file microorganisms-14-01499-s001.zip › microorganisms-4370514-supplementary.pdf]

Supplementary Materials

Supplementary Table S1. Primers used in this study.

Primer	Sequence (5'-3')	Restriction site[a]	Application
azu OHS18_UP_FW	ccgcTCGAGCAGAACGCCAACCA	XhoI	Generation of
azu OHS18_UP_RV	cgggatCCAGGGCCAGCACGGAG	BamHI	pDM4Δazu_{OHS18}
azu OHS18_DOWN_FW	cgggatcCCGCGCTGATGAAGGGTG	BamHI	and screening of Δ*azu*
azu OHS18_DOWN_RV	gctctAGAAAACGACCAGTTGCCGC	XbaI	mutants
azu OHS18_RT_FW	GCATTCCACCGACCAGATGA		PCR validation of
azu OHS18_RT_RV	AGGTAATCCTTGTCGACGCC		Δ*azu* mutants
M13_FW	GTTTTCCCAGTCACGAC		DNA sequencing
M13_RV	AACAGCTATGACCATG		from pBS

[a] The restriction site used for cloning is underlined in the primer sequence.

Supplementary Table S2. List of metabolites identified by NMR-based metabolomic analysis.

**Metabolites**	**Endometabolome**	**Esometabolome**		**HMDB ID**
		Taken up	Released	
(S)-3-Hydroxyisobutyric acid	X		X	HMDB0000023
3-Methyl-2-oxovaleric acid	X			HMDB0000491
Acetic acid	X	X	X	HMDB0000042
Adenosine		X		HMDB0000050
AXP:<br>-Adenosine monophosphate<br>-Adenosine diphosphate<br>-Adenosine triphosphate		X	X	 HMDB0000045<br>HMDB0001341<br>HMDB0000538
Betaine	X	X		HMDB0000043
Cholic acid	X			HMDB0000619
Choline	X	X		HMDB0000097
D-Glucose	X	X	X	HMDB0000122
D-Maltose			X	HMDB0000163
D-Mannose			X	HMDB0000169
D-Ribose			X	HMDB0000283
Formic acid	X	X		HMDB0000142
Fumaric acid	X			HMDB0000134
Glucose 1-phosphate	X			HMDB0001586
Glutamic acid	X	X		HMDB0000148
Glutathione	X			HMDB0000125
Glycerophosphocholine	X	X		HMDB0000086
Glycine	X	X		HMDB0000123
L-Histidine	X	X		HMDB0000177
Hydroxyacetone			X	HMDB0006961
Inosine	X		X	HMDB0000195
L-Isoleucine	X	X		HMDB0000172
Lactic acid	X			HMDB0000190
L-Alanine	X	X		HMDB0000161
L-Arginine		X		HMDB0000517
L-Aspartic acid	X	X		HMDB0000191
L-Leucine	X	X	X	HMDB0000687
L-Threonine			X	HMDB0000167
L-Tryptophan	X		X	HMDB0000929
L-Tyrosine	X		X	HMDB0000158
L-Valine	X		X	HMDB0000883
L-Lysine	X		X	HMDB0000182
L-Methionine	X		X	HMDB0000696
Methylhistidine			X	HMDB0000479
NAD	X			HMDB0000902
NADP	X			HMDB0000217
Niacinamide		X		HMDB0001406
Nicotinic acid		X	X	HMDB0001488
L-Phenylalanine	X		X	HMDB0000159
L-Proline		X		HMDB0000162
Propylene glycol	X		X	HMDB0001881

Pyroglutamic acid		X		HMDB0000267
Succinic acid	X			HMDB0000254
trans-Aconitic acid		X		HMDB0000958
Trehalose		X		HMDB0000975
Uracil	X	X		HMDB0000300
Uridine 5'-monophosphate			X	HMDB0000288
Uridine diphosphate glucose	X			HMDB0000286
Uridine diphosphate-N-acetylglucosamine	X			HMDB0000290

>azu_*Pseudomonas*_sp._OHS18
ATGATTCGCAAGTTTGTTGCTGCCTCCGTGCTGGCCCTGGTCAGTGCGCCGCTATTGGCCGCCGAA
TGTTCGGTAGAGGTGCATTCCACCGACCAGATGACCTTCGATACCAAGGAGATCAAGGTCAGCAAG
AGCTGCAAGACCTTCACCATCGAGCTCAAGCACGTCGGCAACCTGCCCAAGAACGTAATGGGCCAT
AACCTGGTGGTAGCCAAGACCGCGGACGTGCAGGCGATCAGCGCCGATGGCATCGCAGCCGGCGTC
GACAAGGATTACCTGAAGGCTGATGACGCCCGGGTTATCGCCCATACCAAGCTGATTGGCGGCGGC
GAGTCCGACTCGGTGACCTTCGACGTCAGCAAGCTGGATGCTGCCGAGGCCTACCAGTTCTTCTGC
TCCTTCCCCGGCCATGCCGCGCTGATGAAGGGTGCTTTGACCCTGGTCGACTGA

>azurin_*Pseudomonas*_sp._OHS18
MIRKFVAASVLALVSAPLLAAECSVEVHSTDQMTFDTKEIKVSKSCKTFTIELKHVGNLPKNVMGH
NLVVAKTADVQAISADGIAAGVDKDYLKADDARVIAHTKLIGGGESDSVTFDVSKLDAAEAYQFFC
SFPGHAALMKGALTLVD

Alignment

```
                                ....|....| ....|....| ....|....| ....|....| ....|....|
                                        10         20         30         40         50
azurin [P. aeruginosa PAO1]     MLRKLAAVSL LSLLSAPLLA AECSVDIQGN DQMQFNTNAI TVDKSCKQFT
azurin [Pseudomonas sp. OHS18]  MIRKFVAASV LALVSAPLLA AECSVEVHST DQMTFDTKEI KVSKSCKTFT

                                ....|....| ....|....| ....|....| ....|....| ....|....|
                                        60         70         80         90        100
azurin [P. aeruginosa PAO1]     VNLSHPGNLP KNVMGHNWVL STAADMQGVV TDGMASGLDK DYLKPDDSRV
azurin [Pseudomonas sp. OHS18]  IELKHVGNLP KNVMGHNLVV AKTADVQAIS ADGIAAGVDK DYLKADDARV

                                ....|....| ....|....| ....|....| ....|....| ....|....
                                       110        120        130        140
azurin [P. aeruginosa PAO1]     IAHTKLIGSG EKDSVTFDVS KLKEGEQYMF FCTFPGHSAL MKGTLTLK.
azurin [Pseudomonas sp. OHS18]  IAHTKLIGGG ESDSVTFDVS KLDAAEAYQF FCSFPGHAAL MKGALTLVD
```

Supplementary Figure S1. *Pseudomonas* sp. OHS18 azurin coding gene, its amino acid sequence, and its alignment with that of *P. aeruginosa* PA01.

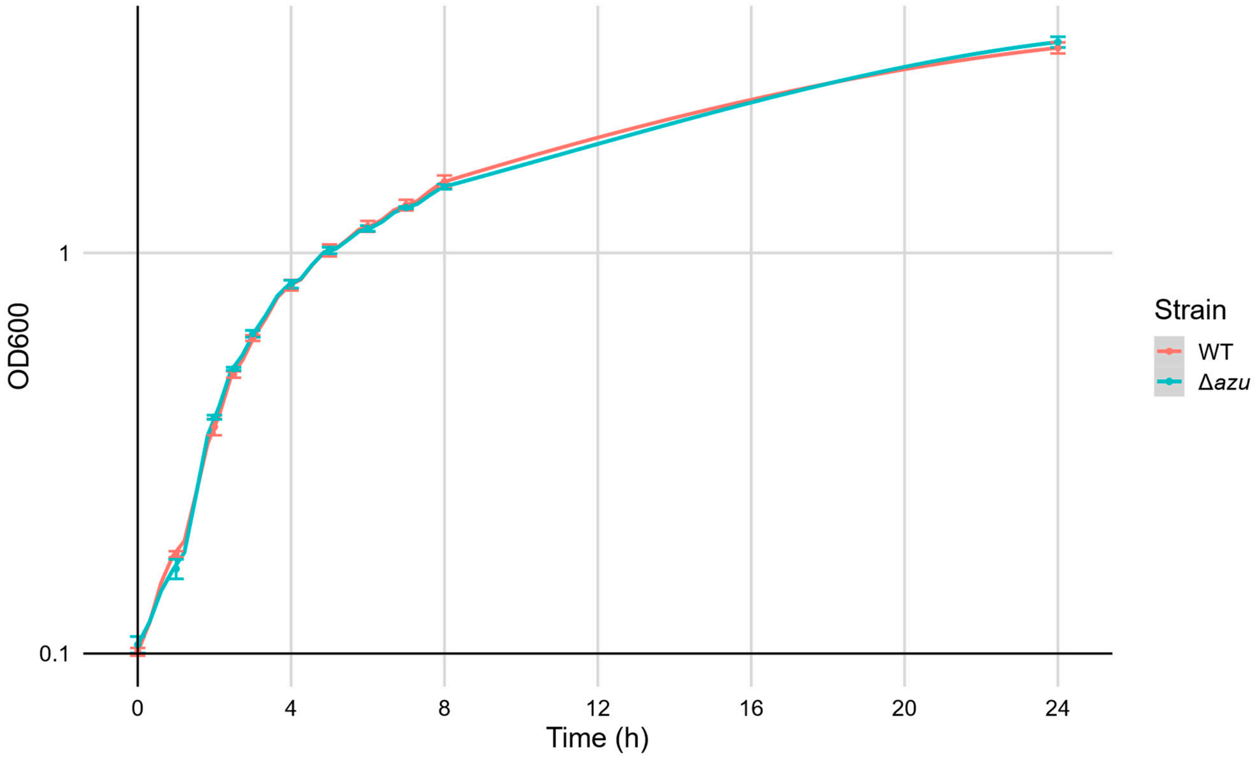

Supplementary Figure S2. Growth kinetics of wild-type (WT) and Δ*azu* strains in Tryptic Soy Broth (TSB).

A single colony of *Pseudomonas* sp. OHS18 wild-type or Δ*azu* strains were inoculated in 10 mL of sterile TSB. Each preculture was used to start a new bacterial culture with an initial OD600=0.1, in a final volume of 200 mL of TSB. The flasks were incubated at 30°C with shaking. Cell growth was monitored by measuring the OD600 every hour using the Infinite 200 PRO multimode reader (Tecan, Männedorf, Switzerland). Each growth curve was performed four times. Curve parameters and graphs were obtained in the R environment using growthcurver and ggplot2 libraries (doi: 10.1186/s12859-016-1016-7; ISBN 978-3-319-24277-4.). This approach fits a logistic growth model to each replicate curve and extracts the following parameters: carrying capacity (K), initial population size (N_0), intrinsic growth rate (r), time to mid-exponential phase (t_mid), doubling time (t_gen), and area under the curve in both log (AUC_l) and empirical (AUC_e) scale, together with the residual standard deviation (σ) as a measure of goodness-of-fit. No statistically significant differences were detected between WT and Δ*azu* strains in any of the fitted growth parameters (Kruskal–Wallis test, $p > 0.05$ for all comparisons), indicating that deletion of azu does not affect growth kinetics under the tested conditions:

**Parameter**	**WT**	**Δ*azu***
**k**	3.2641 ± 0.1107	3.3847 ± 0.11
**n0**	0.2426 ± 0.0096	0.2559 ± 0.0066
**r**	0.3123 ± 0.0131	0.2962 ± 0.0081
**t_mid**	8.1201 ± 0.3357	8.471 ± 0.2552
**t_gen**	2.2313 ± 0.0927	2.3453 ± 0.0625
**auc_l**	51.0114 ± 0.9669	51.6995 ± 0.8813
**auc_e**	44.3403 ± 0.7783	44.8486 ± 0.6485
**sigma**	0.107 ± 0.0068	0.1171 ± 0.0047

Supplementary Table S3. Growth curve parameters of wild-type (WT) and Δ*azu* strains under different culture conditions (TSB + EDTA 0.25 mM, and TSB + EDTA 0.25 mM + $CuSO_4$ 0.1 mM).

	TSB+EDTA 0.25 mM		TSB+EDTA 0.25 mM+CuSO4	
**Parameter***	**WT**	**Δ*azu***	**WT**	**Δ*azu***
**k**	0.1223 ± 0.0196	0.1011 ± 1e-04	0.1489 ± 0.0205	0.1294 ± 0.0035
**r**	0.3257 ± 0.0656	0.4116 ± 0.0185	0.4609 ± 0.0534	0.5371 ± 0.0038
**t_gen**	2.3278 ± 0.505	1.6907 ± 0.0751	1.5494 ± 0.1969	1.2906 ± 0.0091
**t_mid**	10.2404 ± 1.3197	7.6901 ± 0.2707	8.6965 ± 1.2896	6.2716 ± 0.0987
**n0**	0.0057 ± 0.0024	0.0042 ± 6×10^{-4}	0.0031 ± 5×10^{-4}	0.0043 ± 1×10^{-4}
**auc_l**	2.9565 ± 0.3193	2.7509 ± 0.0283	3.8592 ± 0.3352	3.7094 ± 0.0943
**auc_e**	2.9575 ± 0.3172	2.7466 ± 0.0298	3.8878 ± 0.3449	3.7123 ± 0.0975
**sigma**	0.0112 ± 0.003	0.0089 ± 0.001	0.0241 ± 0.0025	0.0168 ± 4×10^{-4}

*carrying capacity (K), initial population size (N_0), intrinsic growth rate (r), time to mid-exponential phase (t_mid), doubling time (t_gen), area under the curve in both log (AUC_l) and empirical (AUC_e) scale, residual standard deviation (σ).

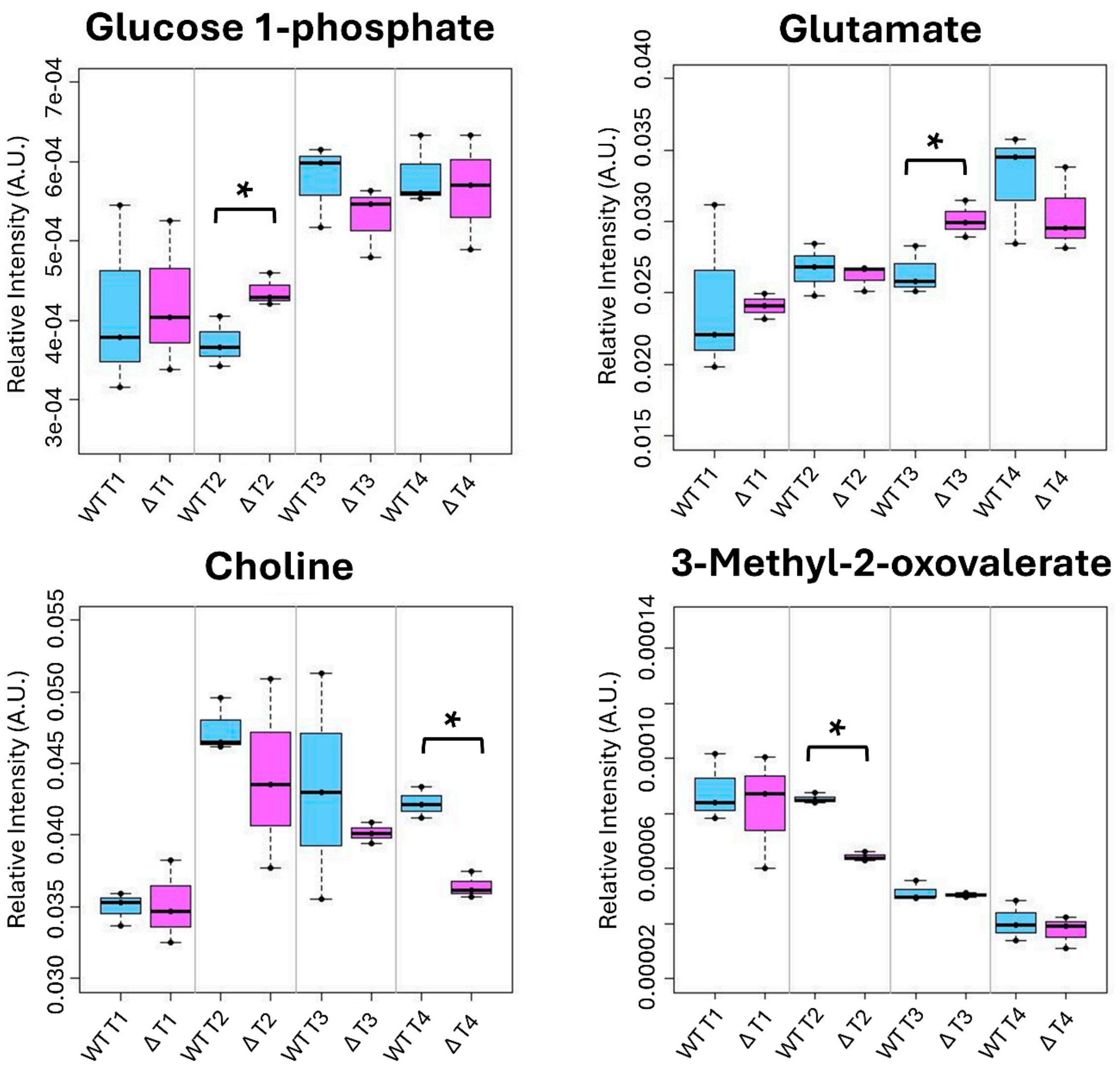

Supplementary Figure S3. Boxplots showing compounds differentially detected in cell lysates. Asterisks indicate statistically significant differences.

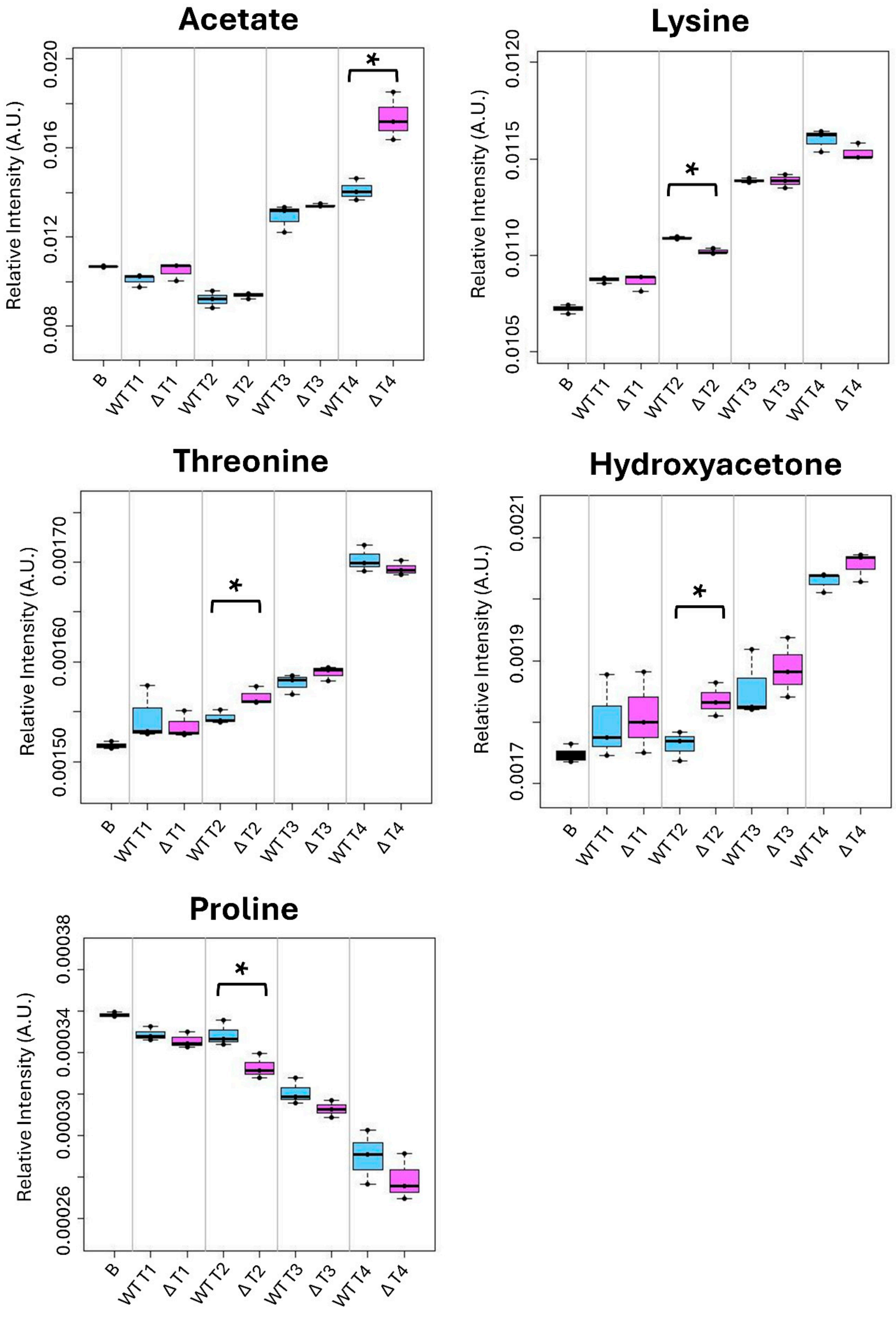

Supplementary Figure S4. Boxplots showing compounds differentially detected in the spent media. Asterisks indicate statistically significant differences.

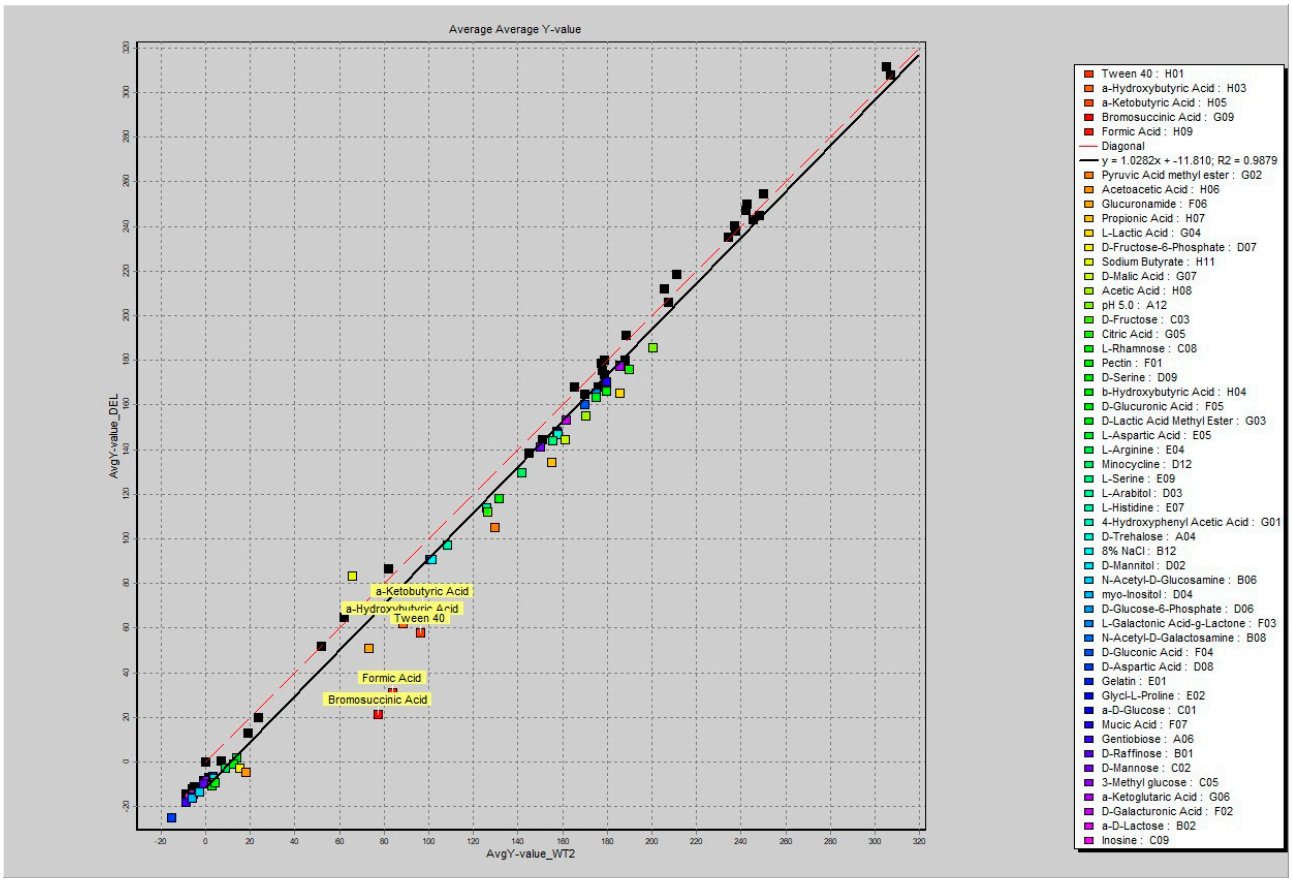

Supplementary Figure S5. Plot showing the average Y-values of the growth curves for each compound in the Biolog GenIII microplate. The X-axis represents the wild-type strain, and the Y-axis represents the mutant. Each compound is color-coded (legend on the right). Compounds that deviate further from the diagonal (highlighted in yellow) are those utilized differently between the two strains.

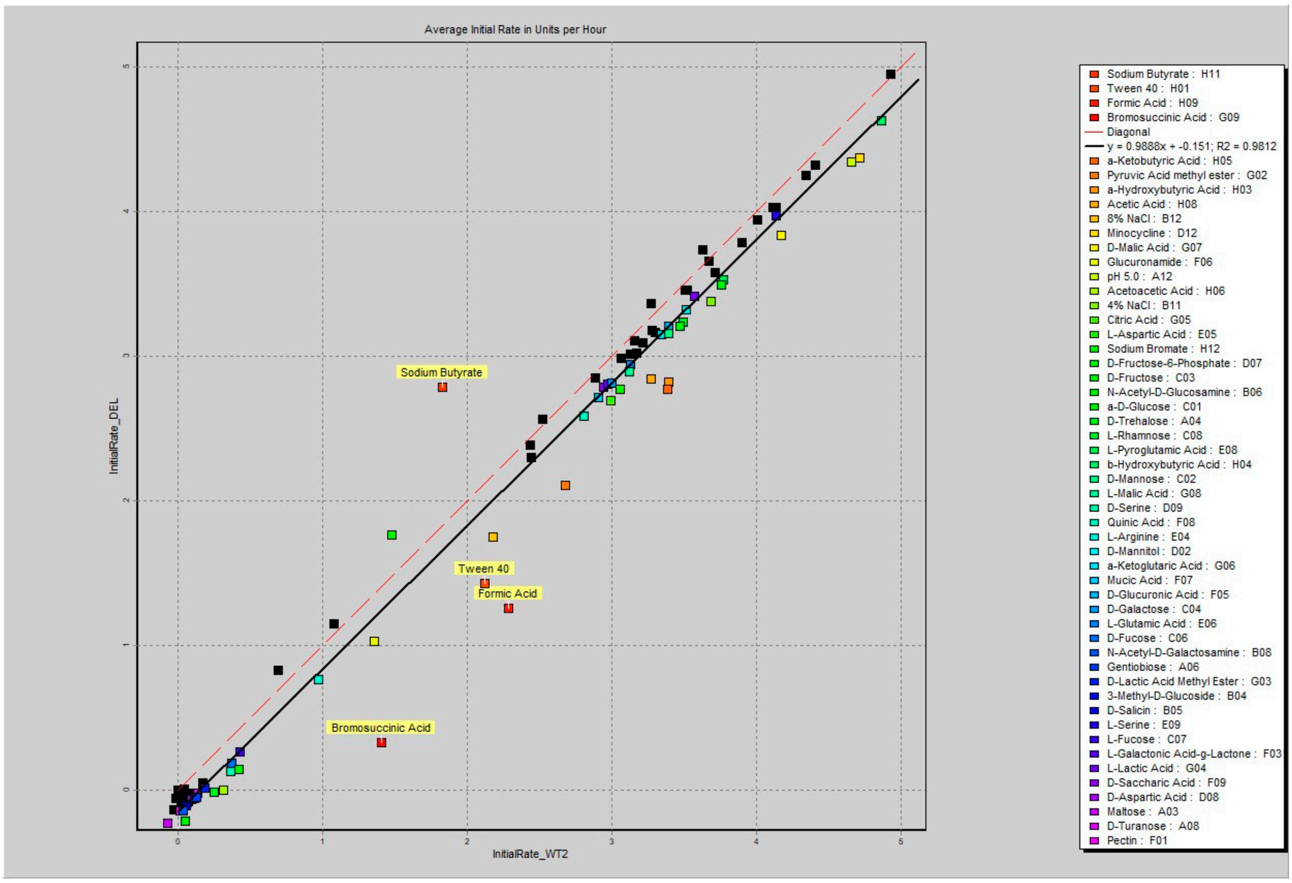

Supplementary Figure S6. Plot showing the average initial rates values (Biolog Units per hour) of the growth curves for each compound in the Biolog GenIII microplate. The X-axis represents the wild-type strain, and the Y-axis represents the mutant. Each compound is color-coded (legend on the right). Compounds that deviate further from the diagonal (highlighted in yellow) are those utilized differently between the two strains.

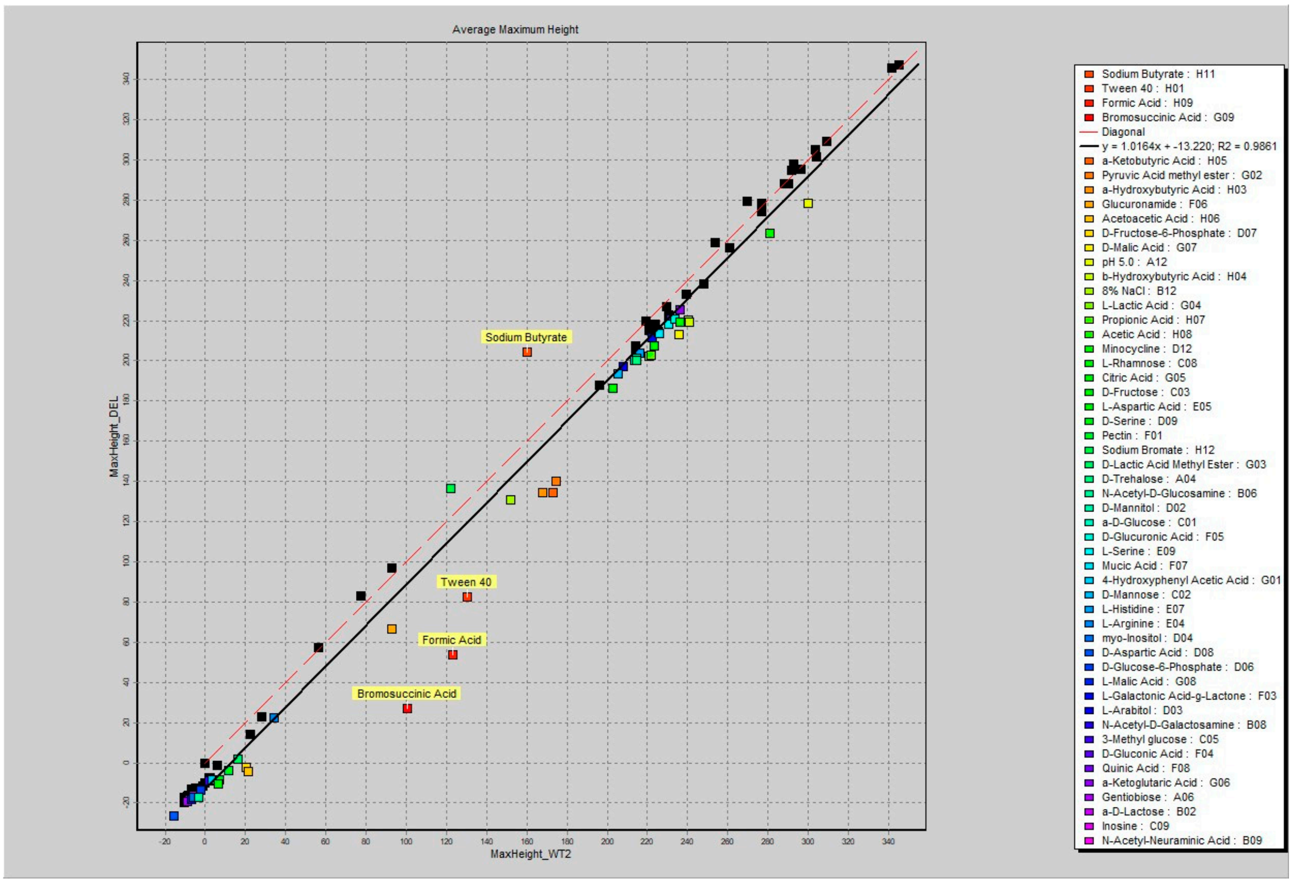

Supplementary Figure S7. Plot showing the average maximum height values (Biolog Units) of the growth curves for each compound in the Biolog GenIII microplate. The X-axis represents the wild-type strain, and the Y-axis represents the mutant. Each compound is color-coded (legend on the right). Compounds that deviate further from the diagonal (highlighted in yellow) are those utilized differently between the two strains.
